# Mental Stress Associated with Shock

**Published:** 1914-12-12

**Authors:** 


### MENTAL STRESS ASSOCIATED WITH SHOCK.

### Some Obscure Results of Violence in Peace and War.

### THE LESSON OF BATTLE SHOCK FOR EXPERT WITNESSES.

The surgical lessons of the war are naturally attracting the attention of the medical profession, and the experiences recorded by various authorities in our societies and journals are full of interest and value. At first sight one would have concluded that among the certainties of surgery was the principle on which wounds ought to be healed, yet, to judge from a recent controversy, there are now acute differences of opinion on the point. These differences seem to be multiplied when details have to be considered, and the merits of carbolic acid and iodine and petrol, not to mention other agents, have excited quite a storm of contradiction. For the present we leave these discussions on one side, having the fairly secure conviction that in an individual case most surgeons would descend from doctrinal disputes, and would be in substantial agreement on the practical measures to be adopted. There are, however, gradually accumulating experiences of the results of violence apart from wounding and other mechanical injury, and though the time has not yet come for subjecting these to an exhaustive analysis it is well that from the outset attention should be directed to them. They touch closely some difficult points of nervous and mental pathology, and are not without significance in relation to the effects of violence as these are at times canvassed by medical witnesses in courts of law.

#### Battle Shock and Mental Breakdown.

One of the groups of cases resulting from the shock of battle and yet unaccompanied by evidences of gross organic damage is marked by mental disturbances of a very serious and anxious order. So frequent, at least relatively, have these been, that, as is well known, a proposal has been made to organise a special hospital for their treatment. With the individual merits of this proposal we are not here concerned. It is sufficient for our present purpose that it has been made and supported by authorities of no inconsiderable weight, and that everyone who has considered the situation agrees in the necessity of some organised scheme of treatment for the cases here in question. The meaning of this position obviously is that violence, and the mental stress associated with violence, are capable of producing in some fashion or other severe and obstinate pathological disturbances in the nervous system, while leaving the integrity of the nervous apparatus unimpaired, at least so far as the ordinary methods of clinical examination are concerned. The cases are not cases of organic nervous disease in the usual acceptation of this term. Apart from the mental state there is nothing to criticise or to condemn. None the less there must be some serious alteration in the functional values of the organic machinery, and this alteration is the result of violence plus the mental influences which accompany violence. The change is there; it is produced in the manner above described, and it is not associated with such organic alterations as display themselves to clinical analysis. All this, it may be remarked, is no new experience. True, but it is a large and impressive display of certain possibilities which may result from violence, and the occurrence of which suggests some measure of caution when the full extent of the consequences of the application of violence to a given individual have to be estimated.

#### Blindness from Shock.

Another series of cases gathered from recent war experience, and having a high degree of interest, consists of men who are found to be blind, though the most exact and skilful examination fails to detect any sign of organic injury, and in time, and under the influence of rest, the patients recover their sight. Here again the strain and shock of battle must have impaired, and impaired seriously, the functional nerve values of some portion of the visual apparatus, though all organic and objective evidence of such impairment escapes entirely the skill of the physician. Mischief has been done, but it is mischief which clinical tests can neither recognise nor identify. The suggestion is obvious that these tests, valuable as everyone knows them to be, are not final and exhaustive, at least in their negative announcements. Hence the conclusion has to be repeated that it is neither safe nor scientific to affirm that the effects of violence are non-existent because no objective evidences of such effects can be found.

#### Violence in Civil Practice.

The truths thus made evident on a large scale in war have, we suggest, some field of application in civil practice. As a result of recent legislative developments the medical practitioner is called upon with increasing frequency to estimate and appraise the pathological consequences of violence. In cases where there is organic evidence of pathological change the position is a comparatively simple one. But where there is no such evidence, while the patient at the same time affirms some functional disability, judgment is often most difficult, and we venture to think that a conclusion adverse to the patient is sometimes too readily presented. That there are fraudulent persons and malingerers no one denies.

But, we now recall, all persons who profess symptoms of disease unaccompanied by clinical signs of organic change are not of this order, and if anyone questions such a proposition there exist to contradict him such war experiences as we have already quoted. Fortunately, the malingerer, for the most part, spoils what might be a good case by exaggeration, and there are instances in which a medical practitioner may speak with the greatest confidence in opposition to professions based on alleged injury.

Malingerers and Medical "Detectives."

There are others in which no medical certainty is possible, and yet it is not altogether uncommon in such cases to hear expert witnesses, both on one side and the other, expressing themselves in terms of the most absolute and unqualified confidence. The position in practice is, we admit, full of difficulties, but two suggestions may be made. The first is that mere absence of organic signs is not a convincing proof of the absence of real suffering and functional disability, as the medical records of the present war make abundantly evident. A second suggestion, advanced with some diffidence, is that the distinction of the malingerer from the genuine complainer cannot be based entirely on a negative clinical examination; something in addition, and more or less of the nature of detective work, is needed, and it is a matter for wonder that in such cases evidence of this order is not more frequently obtained. The duty of the medical practitioner may sometimes include some such scrutiny, but, failing this, let him remember such records as are now coming to us on a comparatively large scale from the seat of war, and not be too ready to announce that all is well merely because the physical results of his clinical examination show no appreciable departure from what is recognised as the normal standard.

